# No Association Between the Home Math Environment and Numerical and Patterning Skills in a Large and Diverse Sample of 5- to 6-year-olds

**DOI:** 10.3389/fpsyg.2020.547626

**Published:** 2020-12-10

**Authors:** Laure De Keyser, Merel Bakker, Sanne Rathé, Nore Wijns, Joke Torbeyns, Lieven Verschaffel, Bert De Smedt

**Affiliations:** Faculty of Psychology and Educational Sciences, KU Leuven, Leuven, Belgium

**Keywords:** home math environment, preschool, mathematics, numeracy, patterning, moderators, gender, SES

## Abstract

Selecting a large and diverse sample of 5–6-year-old preschool children (179 boys and 174 girls; *M*_*age*_ = 70.03 months, *SD*_*age*_ = 3.43), we aimed to extend previous findings on variability in children’s home math environment (i.e., home math activities, parental expectations, and attitudes) and its association with children’s mathematical skills. We operationalized mathematics in a broader way than in previous studies, by considering not only children’s numerical skills but also their patterning skills as integral components of early mathematical development. We investigated the effects of children’s gender and socioeconomic status (SES) on their home math environment, examined the associations between children’s home math environment and their mathematical skills, and verified whether these associations were moderated by children’s gender and/or SES. Parents of 353 children completed a home math environment questionnaire and all children completed measures of their numerical (e.g., object counting) and patterning skills (e.g., extending repeating patterns). Results indicated no effect of children’s gender on their home math environment. There was no effect of SES on the performed home math activities, but small SES differences existed in parents’ math-related expectations and their attitudes. We found no evidence for associations between children’s home math environment and their mathematical skills. Furthermore, there were no moderating effects of gender or SES on these associations. One explanation for these findings might relate to the characteristics of the general preschool system in the country of the present study (Belgium). Future studies should consider the effect of the preschool learning environment because it might explain differences between studies and countries with regard to the home math environment and its association with mathematical skills.

## Introduction

Children’s early mathematical skills at the age of 5 are strong and stable predictors of their later mathematics achievement (e.g., Duncan et al., 2007). Researchers have become increasingly interested in the role of children’s home environment in the development of these early mathematical skills (e.g., LeFevre et al., 2009; Kleemans et al., 2012; Skwarchuk et al., 2014; Blevins-Knabe and Berghout Austin, 2016; Susperreguy et al., 2020b). This is not surprising, given that, for example, Vygotsky (1978) stated in his social development theory that more knowledgeable others can influence and stimulate children’s cognitive skills by means of social interactions, which proceed the process of developmental change. Parents are important agents in young children’s social environments and can therefore create learning opportunities from an early age onwards. In addition, not only parents’ behavior, but also their expectations, beliefs, attitudes, and demographic characteristics (see Eccles, 1993) might impact early child development and achievement (Huntsinger et al., 2000; Blevins-Knabe and Berghout Austin, 2016). Against this background, children’s home math environment as provided by their parents must be considered as a broad construct, including parental activities, expectations, and attitudes. Previous research has revealed large variability in the characteristics of children’s home math environment. These studies have additionally shown that the home environment is positively associated with children’s mathematical skills, even before they start formal schooling in first grade (e.g., Anders et al., 2012; Kleemans et al., 2012; Skwarchuk et al., 2014; Segers et al., 2015; Mutaf-Yildiz et al., 2018a; Napoli and Purpura, 2018; Susperreguy et al., 2020a). However, some studies observed no associations between children’s home math environment and their mathematical skills (Missall et al., 2015) and some studies even reported negative associations (Blevins-Knabe and Musun-Miller, 1996). These observations make the existing body of research less conclusive.

Selecting a large and diverse sample of children, the current study aimed to extend previous findings on the variability in children’s home math environment and on its association with children’s mathematical skills. We operationalized mathematics in a broader way than in most previous studies. We did so by considering not only children’s numerical skills but also their patterning skills as integral components of early mathematical development (e.g., Zippert and Rittle-Johnson, 2020, for a similar discussion). To unravel potential explanations for the observed variability in children’s home math environment, we investigated the effects of children’s gender and socioeconomic status (SES) on their home math environment. We further examined the associations between children’s home math environment and their numerical and patterning skills and investigated whether these associations were moderated by children’s gender and/or SES.

### Variability in Children’s Home Math Environment

Children’s home math environment has been defined as a wide-ranged, multi-componential construct, consisting of various components in children’s home environment that are thought to be beneficial or predictive for children’s mathematical skills (e.g., Niklas and Schneider, 2014; Hart et al., 2016; Napoli and Purpura, 2018). These components include, for example, the math-related activities parents do with their child (e.g., counting, cooking, playing with blocks, creating patterns, and playing games that involve adding or subtracting), as well as parents’ math-related expectations for their children (e.g., mastering mathematical skills such as counting to 100, solving simple additions, reading printed numbers, and multiplying) and their personal attitudes toward mathematics (e.g., considering mathematics as important, enjoying mathematical activities, and considering themselves as good at mathematics) (LeFevre et al., 2010; Missall et al., 2015; Susperreguy et al., 2020b; Zippert and Rittle-Johnson, 2020). Many studies have examined the math-related activities that parents do with their preschoolers, showing large individual differences in the nature and frequency of these activities at this age (e.g., Blevins-Knabe and Musun-Miller, 1996; LeFevre et al., 2009; Missall et al., 2017; Zippert and Rittle-Johnson, 2020). Because socioemotional or affective aspects of the home math environment, such as parental expectations or attitudes, can affect children’s motivation or belief systems toward mathematics, as well as their performance (e.g., Segers et al., 2015; Blevins-Knabe, 2016; see also Eccles, 1993), these affective aspects have also been included in assessments of children’s home math environment. Several studies have shown that these affective variables were positively correlated with the frequency of parent–child math-related activities at home. Thus, parents reporting higher academic expectations for their children or having more positive personal attitudes toward mathematics engage in more math-related activities with their children than parents with lower expectations or less positive attitudes (e.g., LeFevre et al., 2010; Missall et al., 2015; Susperreguy et al., 2020b). However, not all studies have found such associations (Kleemans et al., 2012).

It is important to note that most studies exclusively focused on home math activities and expectations related to early numeracy, and on their relationship with children’s basic numerical skills. Criticizing this limited view on children’s mathematical development, Rittle-Johnson et al. (2015) as well as Zippert and Rittle-Johnson (2020) also considered more complex mathematical skills in their assessment of the home math environment. Specifically, they included items about activities and expectations related to early patterning in their questionnaires. These studies revealed substantial individual differences in the extent to which parents engaged in these patterning activities with their children.

The observed variability in the nature and frequency of various home math environment components raises questions about the factors that might explain this variability. One such factor might be children’s gender. Although gender differences have been the focus of many studies on children’s mathematical development (e.g., Jordan et al., 2006; Stoet and Geary, 2013; Kersey et al., 2018; Bakker et al., 2019; see also Hyde et al., 1990; Lindberg et al., 2010 for meta-analyses), only a limited number of studies has addressed gender differences in children’s home math environment. This body of research has revealed equivocal results. On the one hand, Chang et al. (2011) found that North-American mothers used significantly more overall number-related speech as well as cardinal number speech with boys than with girls. Similarly, Hart et al. (2016) showed that parents of boys reported more home math activities than parents of girls. On the other hand, del Río et al. (2017) found that mothers of Chilean children reported to involve girls significantly more in advanced formal numeracy practices, compared to boys. Similarly, Blevins-Knabe and Musun-Miller (1996) demonstrated that Euro-American mothers reported to significantly engage more in number-related activities, such as counting and singing number songs, with girls than boys. Other studies, however, did not observe gender differences in children’s home math environment (e.g., Jordan et al., 2006; Zippert and Rittle-Johnson, 2020).

A second factor that might explain variability in children’s home math environment is their SES, which refers to “the individual’s or a family’s ranking on a hierarchy according to access to or control over some combination of valued commodities such as wealth, power, and social status” (Sirin, 2005, p. 418). In general, parental education, parental income, and parental occupation are considered as the main indicators of SES (e.g., Sirin, 2005). Parental education has been identified as the most commonly used proxy for SES (Calvo and Bialystok, 2014), and has been used as such in many studies on children’s home math environment. It can be considered as a very stable indicator that is also strongly correlated with other important SES indicators (Sirin, 2005), and it has been identified as the indicator of SES with the strongest associations with children’s educational outcomes (Davis-Kean, 2005; Calvo and Bialystok, 2014).

The studies that investigated the association between children’s SES and their home math environment have yielded, again, inconsistent results, reporting positive, negative, and null relationships. Jordan et al. (2006), for example, observed an association between SES (income) and the frequency of several home math activities, such as talking about numbers and counting objects, and found that middle-income families provided their children with more math-related activities at home, in comparison to low-income households (see also Starkey et al., 1999, as cited in Starkey et al., 2004; Anders et al., 2012; Dearing et al., 2012, for similar results). Likewise, Susperreguy et al. (2020b) reported significantly more shared number-game play as well as counting and arithmetic activities in high SES (parent education) families. Similarly, LeFevre et al. (2010) found positive correlations between SES (parent education) and parental attitudes toward mathematics. Other studies, however, did not find an association between children’s SES (income) and their home math environment (Missall et al., 2015; Hart et al., 2016). Some studies even reported a negative association between SES and math-related activities at home (LeFevre et al., 2010; Niklas and Schneider, 2014). Unfortunately, many studies on children’s home math environment were done in small samples and in samples that were homogeneous in terms of children’s SES (for exceptions see Anders et al., 2012; Niklas and Schneider, 2014; Susperreguy et al., 2020a). To more thoroughly examine the effects of children’s SES on their home math environment, it is critical that samples are sufficiently large and diverse in terms of SES. This is also essential to further understand the association between children’s home math environment and their mathematical skills, as we will document below.

### Associations Between Children’s Home Math Environment and Their Mathematical Skills

The literature on the association between children’s home math environment and their mathematical skills is, again, equivocal. Various studies provide support for positive associations between the frequency of home math-related activities and children’s numerical skills (e.g., Kleemans et al., 2012; Skwarchuk et al., 2014; Segers et al., 2015; Mutaf-Yildiz et al., 2018a; Napoli and Purpura, 2018; Susperreguy et al., 2020a; Zippert and Rittle-Johnson, 2020). Similar positive associations have been observed between parents’ math-related expectations and attitudes, and children’s numerical skills. Segers et al. (2015), for example, found that parents’ numeracy expectations for their children were positively associated with children’s early numerical skills. In addition, LeFevre et al. (2010) found that parental math-related attitudes (e.g., I enjoy mathematics) predicted children’s early numeracy outcomes. Other studies, however, reported negative associations. For example, Skwarchuk (2009) observed that parents’ engagement in basic numerical activities (e.g., counting objects and reciting numerals) was negatively related to 3- to 5-year-olds’ mathematical ability (see also Blevins-Knabe and Musun-Miller, 1996). Some studies, however, reported no association between children’s home math environment and their numerical (Blevins-Knabe et al., 2000; Missall et al., 2015) or patterning skills (Zippert and Rittle-Johnson, 2020).

These varying results between studies on the association between children’s home math environment and their mathematical skills might be explained by moderating variables. Against the background of the above-reviewed studies on the variability in children’s home math environment, gender and SES might be such moderating variables. We therefore examined the moderating effects of gender and SES on the association between children’s home math environment and their mathematical skills. The examination of these moderating effects requires a large and diverse sample, for which reason we selected a large number of children who came from a diversity of SES backgrounds.

### The Current Study

We investigated children’s home math environment and its association with children’s mathematical skills in the specific educational context of Flanders (Belgium). In the Flemish educational system, nearly all children (98%) attend fully government subsidized preschool (age 2.5–6 years), which includes a playful non-formal introduction in various mathematical domains. We extended the existing literature through a more thorough assessment of children’s math-related home experiences and early mathematical skills by focusing not only on early numeracy but also on patterning, which can also be considered as a critical aspect of preschool children’s mathematical development (e.g., Klein and Starkey, 2004; Clements and Sarama, 2014; Rittle-Johnson et al., 2015; Wijns et al., 2019b; Zippert and Rittle-Johnson, 2020). We also extended the previous literature on the role of SES and gender in children’s home math environment by examining this in a diverse and large sample. Moreover, in most home math environment studies only frequentist analytical approaches have been used. We extended the existing literature by additionally using Bayesian analyses.

Specifically, we investigated the home math environment in children who were enrolled in the third year of preschool (5- to 6-year-olds). Via a parent questionnaire, which was similar to the one applied in previous studies (e.g., LeFevre et al., 2009; Kleemans et al., 2012; Mutaf-Yildiz et al., 2018b; Rathé et al., 2020), we assessed parents’ home math activities as well as their expectations and attitudes toward mathematics, and included questions on both number and patterning. Children’s SES was determined by the mother’s educational level, as parental educational level has been shown to be a critical predictor of children’s achievement (e.g., Davis-Kean, 2005). Previous research has documented high correlations between the educational levels of mothers and fathers (Eika et al., 2019) and has shown that the effects of mothers’ and fathers’ education appear to be comparable (Marks et al., 2000). Due to the specific role mothers often occupy in children’s life (Marks et al., 2000), we primarily focused on the educational level of the mother. All children completed two measures that were designed to assess their mathematical skills. The first measure focused on children’s numerical skills and consisted of a set of tasks (e.g., counting, number comparison, number identification, and arithmetic) that have been frequently used to investigate children’s early numerical skills (e.g., Jordan et al., 2006; Purpura and Lonigan, 2013) and that overlap with conventional standardized tests, such as TEMA. Similar measures have often been used in research on children’s home math environment at this age (e.g., LeFevre et al., 2009; Kleemans et al., 2012; Susperreguy et al., 2020b). The second measure assessed children’s repeating patterning skills, and this measure was highly similar to the measures that have been used in previous research on preschoolers’ patterning skills (e.g., Rittle-Johnson et al., 2015; Zippert and Rittle-Johnson, 2020).

We posited three research questions: (1) Are there effects of children’s gender and/or SES on the home math environment? (2) Is there evidence for an association between children’s home math environment and their mathematical skills, i.e., numerical and patterning skills? (3) Is the association between children’s home math environment and their mathematical skills moderated by their gender and/or SES? In view of the abovementioned conflicting findings reported in the literature, no directional hypotheses were put forward.

## Materials and Methods

### Participants

The current study reports on data that have been collected in a large-scale longitudinal research project on young children’s core mathematical competencies^1^. The study was approved by the Social and Societal Ethics Committee of KU Leuven (G-2016 07 591). This ongoing research project follows a cohort of 410 children from their second year of preschool, when they were 4-5 years old (2017), until the third year of elementary school (2021). Data of the first year of this longitudinal study have already been published (Bakker et al., 2019; Wijns et al., 2019a,b). The current study reports on data that were collected in the second year of the research project, during which children were in their third and final preschool year. In that year, data on children’s home math environment as well as on their numerical and patterning skills were collected.

The original sample was selected by means of a stratified cluster strategy to obtain a sample with an SES distribution that is representative for the Flemish context. Stratification was based on the so-called school-level SES, which is determined by the relative number of children receiving a study allowance and the relative number of children whose mother did not obtain a secondary school certificate. Schools were classified into quartiles that range from having children with predominantly low (Q1) to high (Q4) SES. We recruited 17 schools, which were equally distributed across the four SES quartiles. All children attending the second year of preschool in these schools (*N* = 517) were eligible to participate. Parents of 410/517 children (*Q1* = 112, *Q2* = 103, *Q3* = 94, *Q4* = 101) gave written informed consent for participation in the research project (Response rate = 79%).

The current sample, i.e., participants in the second year of our longitudinal study, consisted of 389 children. Parents of the participating children were asked to complete the parent questionnaire, when their children were enrolled in the first semester (fall 2017) of their third year of preschool. We received 363/389 questionnaires (Response rate = 93%). Children’s numerical and patterning skills were measured later in the school year (spring 2018). At this point, data of 10 children were missing due to changing schools or technical problems during data collection, and the data of these 10 children were further discarded. The final sample consisted of 353 children, including 179 boys (*M*_*age*_ = 70.03 months, *SD*_*age*_ = 3.36) and 174 girls (*M*_*age*_ = 70.01 months, *SD*_*age*_ = 3.51). Most of these children were Belgian (92%) and had Dutch as their mother tongue (82%). All participating children were sufficiently proficient in Dutch to understand and complete the tasks they were asked to perform.

Most respondents to the parent questionnaire were mothers (269/353 or 76%). The respondents in the remaining cases were fathers (75/353 or 21%), grandparents (2/353 or 0.6%) or unknown (7/353 or 2%). Of the 353 respondents, 51% was in the 35–40 age group. Information on the educational level of 348/353 mothers (99%) and 322/335 fathers (96%) was available. These data showed that the participating children came from a diverse range of socioeconomic backgrounds. The educational level of 41 (12%), 95 (27%), 79 (23%), and 133 (38%) mothers was distributed across the low (= no education, primary education or lower secondary level education), below-average (= upper secondary level education), above-average (= professional bachelor degree) and high (= academic bachelor’s degree, master’s degree or doctoral degree) SES categories, respectively. In addition, the educational level of 51 (16%), 97 (30%), 58 (18%), and 116 (36%) fathers was distributed across the low (= no education, primary education or lower secondary level education), below-average (= upper secondary level education), above-average (= professional bachelor degree) and high (= academic bachelor’s degree, master’s degree or doctoral degree) SES categories, respectively. Most parents were Belgian (85%) and had Dutch as their mother tongue (75%).

As stated above, the educational context in which the current data were collected is characterized by a 3-year non-compulsory fully government subsidized preschool system, which starts at the age of 2.5 years and guarantees freedom of school choice. As documented by the Economist Intelligence Unit (2012), Belgium scores very high on several indicators of the Starting Well Index: quality (e.g., teacher quality and training, setting out curriculum guidelines and standards, ensuring parental engagement; 5th place of 45 countries), availability (preschool enrolment ratio and legal right to preschool education, 1st place of 45 countries) and affordability of the provided preschool program (e.g., government pre-primary education spending and subsidies for underprivileged families, 5th place of 45 countries). Moreover, preschool quality is homogeneous in Belgium. This is a result of, for example, the regulations regarding educational standards, which are designed and supervised by the federal government. Because nearly all children (98%) attend preschool, almost all children receive a specific, high-quality, albeit non-formal, introduction to several mathematical domains (e.g., counting, measuring, and patterning) from an early age at school. Schools are free to develop their own curricula – including their math-related activities – but they have to aim for specific core learning goals that are specified by the government (Agentschap voor Hoger Onderwijs, Volwassenenonderwijs, Kwalificaties en Studietoelagen, n.d.). The section on math-related preschool learning focuses on various mathematical domains and consists of core learning goals, such as counting up to five objects, comparing quantities, and extending patterns.

### Measures

#### Home Math Environment

The home math environment was measured via a paper-and-pencil questionnaire that included questions pertaining to the frequency of parent–child mathematical home activities (7 items), parents’ expectations regarding their child’s mathematical skills (10 items), as well as their attitudes toward mathematics (3 items). This questionnaire was based on Dutch adaptations of the original questionnaire of LeFevre et al. (2009) by Kleemans et al. (2012) and Rathé et al. (2020). An overview of the items in this questionnaire is included in Supplementary Appendix A. The frequency of home math activities had to be indicated on a 5-point Likert scale ranging from never (0) to every day (4). Parents were asked how often they engaged with their child in several math-related activities, such as counting or elementary calculations during daily activities and creating patterns with concrete materials. Parental expectations were measured on a 4-point Likert scale, ranging from not at all important (0) to very important (3), in which they had to indicate how important it was for them that their child masters certain competencies at the start of first grade (e.g., reciting the number sequence up to 10 and extending a pattern). For the last section, parents had to indicate their personal attitudes toward mathematics (e.g., mathematics is important) on a 5-point Likert scale, ranging from completely disagree (0) to completely agree (4). For every participant, an average score was calculated for the three subscales separately. The questionnaire had sufficient reliability with Cronbach’s alpha of 0.79, 0.89, and 0.74 for the activities, expectations, and attitudes items, respectively.

#### Socioeconomic Status (SES)

Children’s SES was also investigated via the parent questionnaire. Respondents had to indicate the highest educational level of both mother and father. These responses were then categorized into four SES categories: low = no education, primary education or lower secondary level education; below-average = upper secondary level education; above-average = professional bachelor degree; high = academic bachelor’s degree, master’s degree or doctoral degree. Analyses were based on the SES category of the mother. When data on mother’s SES category were missing, we checked whether we could use the category of the father. However, for all participants, when data on the educational level of the mother were missing, information on the educational level of the father was also not available.

#### Numerical Skills

This measure was highly similar to the materials used in Bakker et al. (2019) and Wijns et al. (2019a, b), which reported on the first year of our longitudinal research project, when children were in their second preschool year (age 4–5). The measure comprised the following tasks: verbal counting (i.e., “Count as high as you can”; 1 item), verbal arithmetic (i.e., “I put N stones in a box, I add/subtract M stones, how many stones are in my box now?”; 8 items), object counting (i.e., “Put N stones on the table.”; 8 items), Arabic numeral recognition (i.e., “Which number is this?”; 30 items), number order (i.e., “Which number comes before and after N?”; 8 items), symbolic calculation (i.e., “Look at the card, how much do you get when you add/subtract N and M?”; 8 items), symbolic number comparison (i.e., “Which of the two numbers is the largest?”; 12 items), non-symbolic number comparison (i.e., “Which dot array has more dots?”; 12 items), and dot enumeration (i.e., “How many dots do you see?”; 18 items). Data were standardized per task, and the average of these standardized scores was used as dependent variable in our analyses. The reliability of this score was high (α = 0.94).

#### Patterning Skills

Children’s repeating patterning skills were assessed by means of the same materials as used by Wijns et al. (2019a). The measure consisted of 18 items that focused on three repeating patterning activities, namely extending (i.e., “Which figure has to be placed in the empty spot at the end of the row?”; 6 items), translating (i.e., “Make the same pattern as above with these figures.”; 6 items), and identifying (i.e., “Look at this row, remember the pattern, and reconstruct it.”; 6 items) a pattern. All patterns were spatial to minimize the impact of verbal abilities. For each task, data were standardized and the average of the standardized scores was used as dependent variable in our analyses. The reliability of this measure was good (*α* = 0.80).

### Procedure

Parents completed the home math environment questionnaire in the fall of 2017, when children were enrolled in their first semester of the third year of preschool. The questionnaire was available in multiple languages (i.e., Dutch, French, and English) and was distributed via the schools of the children. Data on children’s numerical and patterning skills were collected 6 months later, in the spring of 2018. Children were tested in two individual sessions of approximately 30 min, which took place in a quiet room at the children’s school. Patterning skills were measured in the first session and numerical skills were measured in the second session. The test sessions took place on two different days and all tasks were administered in the same order to each child. The child assessments were conducted by the Ph.D. students working on the longitudinal research project and by several research assistants. All experimenters followed extensive training sessions to get familiar with the tasks and to ensure data quality by minimizing tester effects.

### Analytic Approach

We employed both frequentist and Bayesian statistics to analyze our data with the IBM SPSS Statistics 25 (IBM Corp., 2017), IBM SPSS Statistics 26 (IBM Corp., 2019), PROCESS Macro for SPSS 3.5 (Hayes, 2020), and JASP 0.11.1 software (JASP Team, 2019). Against the background of conflicting evidence on the association between the home math environment and mathematical skills, Bayesian analyses were added because they allowed us to quantify the evidence in support of the alternative hypothesis (H_1_) compared to the null hypothesis (H_0_). This is expressed in the Bayes Factor (BF_10_), which indicates the ratio of the evidence in support of H_1_ over H_0_. For example, BF_10_ = 15 indicates that the data are 15 times more likely under the alternative hypothesis than under the null hypothesis. These Bayes Factors are a continuous index of support for one or another hypothesis, although there are some conventions for interpreting the size of these factors (Andraszewicz et al., 2015). Specifically, BF_10_ = 1 provides no evidence either way, whereas BF_10_ > 1 provides anecdotal evidence, BF_10_ > 3 provides moderate evidence, BF_10_ > 10 provides strong evidence, BF_10_ > 30 provides very strong evidence, and BF_10_ > 100 provides decisive evidence for the alternative hypothesis. On the other hand, BF_10_ < 1 provides anecdotal evidence, BF_10_ < 1/3 provides moderate evidence, BF_10_ < 1/10 provides strong evidence, BF_10_ < 1/30 provides very strong evidence, and BF_10_ < 1/100 provides decisive evidence for the null hypothesis.

Pearson correlations were calculated to examine the associations between the different variables under study. The Bayesian analyses used the default prior (stretched beta with prior of 1). Effects of child gender and SES on children’s home math environment were investigated via *t*-tests and ANOVAs, respectively, the latter of which were corrected for multiple comparisons via Bonferroni *post hoc* tests. The Bayesian *t*-tests used the default Cauchy distribution centered around 0 with a width of 0.707 and the Bayesian ANOVAs used the default prior of *r* scale = 0.5.

We examined the moderating effects of child gender and SES on the relationship between children’s home math environment and their numerical and patterning skills by calculating regression models using the PROCESS Macro for SPSS 3.5 (Hayes, 2020). In these models, numerical and patterning skills were predicted by home math environment variables, child gender or SES, and, critically, by the interaction between a particular home math environment variable (activities/expectations/attitudes) and child gender or SES. This interaction indicates whether moderation occurred or not. All home math environment factors were centered before they were included in the models. We used Helmert coding when including the multicategorical SES factor in our regression models, because of the ordinal dimension of this variable (Hayes, 2017). Using this coding system, the mean of each level of the SES variable, starting from the lowest SES category, is compared with the mean of all the subsequent SES levels (low versus below-average, above-average, and high SES; below-average versus above-average and high SES; above-average versus high SES) (UCLA Statistical Consulting Group, 2020). Lastly, if the interaction of a home math environment variable and gender or SES was significant, we further examined the association between numerical or patterning skills and home math factors for each level of the moderator.

All abovementioned analyses were repeated controlling for children’s age. This did not change any of our findings.

## Results

Descriptive statistics of all administered measures are presented in Table 1. A detailed description per item of the home math measure is included in Supplementary Appendix B. There were some missing data on the home environment questionnaire, due to parents skipping one or multiple items. Pairwise deletion was used when substantial information (i.e., non-response on more than half of the items of a subscale) was missing on the activities, expectations, and attitudes scales, for which reason the sample size varied across subscales. The rate of missing data was less than 5% for all items and data were missing completely at random [Little’s MCAR test χ*^2^*(471) = 466.52, *p* = 0.550].

**TABLE 1:** Descriptive statistics of the administered home math environment and outcome measures.

	Informant	*n*	*M*	Min	Max	*SD*
**Home math environment**						
Activities (0–4 scale)	Parent	351	1.81	0.00	3.71	0.69
Expectations (0–3 scale)	Parent	347	2.03	0.20	3.00	0.54
Attitudes (0–4 scale)	Parent	348	2.92	0.00	4.00	0.77
Numerical skills (standardized composite)	Child	353	0.04	−1.91	1.37	0.68
Patterning skills (standardized composite)	Child	353	0.05	−2.09	1.12	0.73

There was considerable variability across the different activities, expectations, and attitudes reported by the parents and for most of the items the full range of response options was used. The home math activities that occurred most frequently (Supplementary Appendix B) were “counting or elementary calculations during daily activities (e.g., counting the number of apples during cooking)” (*M* = 2.57) and “attending to written numerals during daily activities (e.g., cooking)” (*M* = 2.13). The two patterning-related activities were performed the least frequently (*M* = 1.17 and 1.36). Parents reported to have the highest expectations for their children on “reciting the number sequence up to 10 (e.g., 1, 2, 3, 4, …)” (*M* = 2.68) and “counting up to 10 objects (e.g., counting 3 candies)” (*M* = 2.57), and the lowest expectations on “solving sums up to 10 (e.g., 5 + 4)” (*M* = 1.23). Frequentist correlation analyses showed significant correlations between all three home math environment components. Parents who engaged in more math-related activities had higher math-related expectations for their children, and the Bayesian evidence for this association was decisive [*r*(345) = 0.39, *p* < 0.001, BF_10_ > 100]. There were small positive associations between parents’ attitudes and their activities [*r*(346) = 0.12, *p* = 0.024, BF_10_ = 0.86] and expectations [*r*(342) = 0.12, *p* = 0.023, BF_10_ = 0.90], but the Bayes factors were close to 1 suggesting neither evidence in favor nor against an association.

### Effects of Gender

As indicated in Table 2, there were no gender differences in any of the home math-related variables. The Bayesian analyses provided substantial evidence in favor of the null hypothesis of gender equality in children’s home math experiences (BF_10_ < 1/3).

**TABLE 2:** Gender differences in the home math environment and children’s numerical and patterning skills.

Variable	Girls	Boys					
	*M (SD)*	*M (SD)*	*df*	*t*	*p*	Cohen’s *d*	BF_10_
**Home math**							
Activities	1.76	1.85	349	1.22	0.225	0.13	0.24
	(0.66)	(0.72)					
*n*	173	178					
Expectations	2.00	2.06	345	0.93	0.351	0.10	0.18
	(0.54)	(0.54)					
*n*	172	175					
Attitudes	2.89	2.95	346	0.66	0.507	0.07	0.15
	(0.79)	(0.74)					
*n*	172	176					
Numerical skills	−0.02	0.09	351	1.61	0.107	0.17	0.41
	(0.65)	(0.70)					
*n*	174	179					
Patterning skills	0.14	−0.04	351	−2.37	0.018	−0.25	1.73
	(0.72)	(0.74)					
*n*	174	179					

Although not the primary focus of this study, we additionally explored gender differences in children’s numerical and patterning skills. There were no gender differences in numerical skills, yet a small difference favoring girls was observed for children’s patterning skills, but the evidence was only anecdotal (BF_10_ = 1.73).

### Effects of SES

There were no differences in the frequency of parent–child home math activities between the SES groups (Table 3). The Bayes Factors indicated strong evidence in favor of this null hypothesis (BF_10_ < 1/3). The SES groups differed significantly in terms of parental math-related child expectations: parents with low SES had significantly higher expectations than those with above-average (*p* = 0.008; *d* = 0.67) and high SES (*p* = 0.005; *d* = 0.57). The evidence for SES differences in parental expectations was moderate (BF_10_ = 9.64). Parents of children from the SES groups also differed significantly in their math attitudes and the evidence for this hypothesis was strong (BF_10_ = 18.57). Follow-up comparisons revealed significant differences between the attitudes from parents of the high SES group and both the above-average (*p* = 0.027; *d* = −0.40) and below-average SES group (*p* = 0.002; *d* = −0.51), with the former having more positive attitudes than the latter ones.

**TABLE 3:** SES differences in the home math environment and children’s numerical and patterning skills.

	Low	Below-average	Above- average	High					
						
Variable	*M (SD)*	*M (SD)*	*M (SD)*	*M (SD)*	*df*	*F*	*p*	η^2^	BF_10_
Home math	1.65	1.90	1.75	1.82					
activities	(0.88)	(0.66)	(0.69)	(0.65)	3, 342	1.51	0.212	0.01	0.10
*n*	39	95	79	133					
	2.28	2.11	1.94	1.95					
Expectations	(0.50)	(0.48)	(0.50)	(0.59)	3, 338	5.16	0.002	0.04	9.64
*n*	40	93	78	131					
Attitudes	2.94 (0.62)	2.74 (0.76)	2.80 (0.85)	3.11 (0.72)	3, 339	5.33	0.001	0.05	18.57
*n*	38	93	79	133					
Numerical skills	−0.24 (0.70)	−0.15 (0.70)	0.11 (0.62)	0.22 (0.63)	3, 344	8.90	<0.001	0.07	1503.58
*n*	41	95	79	133					
Patterning skills	−0.21 (0.76)	−0.14 (0.80)	0.17 (0.61)	0.22 (0.70)	3, 344	7.14	<0.001	0.06	152.63
*n*	41	95	79	133					

*Mothers’ educational level was used as a proxy of children’s SES.*

Although it was not the primary focus of this study, we additionally explored SES differences in children’s numerical and patterning abilities. Significant differences between the SES groups were observed for both numerical and patterning abilities, and these differences were decisive (BF_10_ > 100). Follow-up comparisons revealed that children from the low SES group performed significantly more poorly on both numerical and patterning tasks than children from the above-average or high SES group (*p* < 0.05). Children with below-average SES showed poorer numerical abilities than children of the high SES group and poorer patterning abilities than children of both the above-average and high SES group (*p* < 0.05).

### Associations Between the Home Math Environment and Children’s Mathematical Skills

We subsequently analyzed the associations between children’s home math environment and their numerical and patterning skills (Table 4). Parent–child math activities and parental expectations were not correlated with children’s numerical skills. The Bayesian approach even indicated strong evidence for the absence of these correlations. A small positive correlation between parents’ math attitudes and children’s numerical skills was observed, yet the evidence for this association was anecdotal. No associations were observed between the three components of children’s home math environment and their patterning skills, and the evidence for an absence of these associations was moderate to strong. Children’s numerical and patterning skills were highly correlated [*r*(352) = 0.59, *p* < 0.001, BF_10_ > 100] and this remained when children’s age was additionally controlled for.

**TABLE 4:** Correlations between children’s home math environment, numerical skills, and patterning skills.

Home math environment	Numerical skills				Patterning skills			
	*df*	*r*	*p*	BF_10_	*df*	*r*	*p*	BF_10_
Activities	349	0.02	0.751	0.07	349	−0.09	0.109	0.24
Expectations	345	0.04	0.449	0.09	345	−0.04	0.469	0.09
Attitudes	346	0.14	0.011	1.70	346	0.01	0.894	0.07

### Moderating Effects of Gender and SES

Additionally, we verified whether the associations between children’s home math environment and their numerical as well as patterning skills were moderated by their gender or SES. This was done via regression models in which numerical and patterning skills were predicted by home math environment components, gender or SES, and, critically, by the interaction between a particular home math environment component (activities, expectations, or attitudes) and gender or SES. These interactions are reported in Tables 5, 6 and indicate whether moderation occurred or not (see Supplementary Appendix C for full details of the regression analyses). The analyses revealed that neither gender nor SES moderated the associations between children’s home math environment and their numerical (Table 5) and patterning (Table 6) skills, given that all interaction effects were non-significant.

**TABLE 5:** Moderation analyses of the association between children’s home math environment and their numerical skills.

	*B*	*SE*	*t*	*p*
**Gender**				
Activities*Gender	−0.06	0.11	−0.61	0.545
Expectations*Gender	−0.25	0.14	−1.84	0.067
Attitudes*Gender	0.00	0.09	−0.01	0.990
**SES**				
Activities*SES-1 (1 vs. 2, 3, 4)	0.11	0.13	0.85	0.398
Activities*SES-2 (2 vs. 3, 4)	−0.09	0.12	−0.75	0.454
Activities*SES-3 (3 vs. 4)	−0.05	0.14	−0.33	0.744
Expectations*SES-1 (1 vs. 2, 3, 4)	0.03	0.22	0.12	0.904
Expectations*SES-2 (2 vs. 3, 4)	−0.28	0.17	−1.67	0.095
Expectations*SES-3 (3 vs. 4)	0.10	0.18	0.58	0.564
Attitudes*SES-1 (1 vs. 2, 3, 4)	−0.14	0.18	−0.80	0.424
Attitudes*SES-2 (2 vs. 3, 4)	−0.07	0.11	−0.63	0.527
Attitudes*SES-3 (3 vs. 4)	−0.15	0.12	−1.31	0.192

*Coefficients correspond to the interaction term between the moderator (gender or SES) and components of the home math environment. Analyses including SES as a predictor were performed using Helmert coding (1 = low SES; 2 = below-average SES; 3 = above-average SES; 4 = high SES).*

**TABLE 6:** Moderation analyses of the association between children’s home math environment and their patterning skills.

	*B*	*SE*	*t*	*p*
**Gender**				
Activities*Gender	−0.01	0.11	−0.05	0.963
Expectations*Gender	−0.11	0.15	−0.72	0.474
Attitudes*Gender	0.14	0.10	1.33	0.184
**SES**				
Activities*SES-1 (1 vs. 2, 3, 4)	0.16	0.15	1.12	0.264
Activities*SES-2 (2 vs. 3, 4)	0.19	0.13	1.40	0.162
Activities*SES-3 (3 vs. 4)	−0.08	0.15	−0.55	0.585
Expectations*SES-1 (1 vs. 2, 3, 4)	−0.10	0.24	−0.40	0.694
Expectations*SES-2 (2 vs. 3, 4)	−0.01	0.19	−0.06	0.955
Expectations*SES-3 (3 vs. 4)	0.04	0.20	0.22	0.824
Attitudes*SES-1 (1 vs. 2, 3, 4)	−0.12	0.20	−0.62	0.538
Attitudes*SES-2 (2 vs. 3, 4)	0.13	0.12	1.08	0.282
Attitudes*SES-3 (3 vs. 4)	−0.13	0.13	−1.03	0.302

*Coefficients correspond to the interaction term between the moderator (gender or SES) and components of the home math environment. Analyses including SES as a predictor were performed using Helmert coding (1 = low SES; 2 = below-average SES; 3 = above-average SES; 4 = high SES).*

## Discussion

Social interactions with more knowledgeable others can stimulate children’s early cognitive development (Vygotsky, 1978). This suggests that parents might be able to create important learning opportunities for their children even before the start of formal schooling and confirms the importance of children’s home environment. Not only the activities parents engage in with their children, but also their expectations, beliefs, and attitudes can influence children’s early cognitive outcomes (e.g., Eccles, 1993; Huntsinger et al., 2000). Against this background, children’s home math environment as provided by their parents has been considered as a broad construct, including parental activities, expectations, and attitudes. We extended the existing body of literature on children’s home math environment and its association with their mathematical performance by operationalizing mathematics in a more comprehensive way than previous work, considering not only number but also patterning as critical domains of children’s home math environment and of their early mathematical development. In order to more carefully investigate the broad spectrum of individual differences, we specifically recruited a large, representative, and diverse sample of children. This allowed us to analyze the effects of children’s gender and SES on the variability in children’s home math environment, and even more critically, whether these variables moderated the associations between children’s home math environment and their mathematical skills. Understanding such moderating effects might explain why previous studies have reported conflicting findings on these associations.

Our data revealed that, despite substantial variability in the home math environment of Flemish children aged 5–6 years old, no gender differences in children’s home math environment were observed. There were also no differences between the SES groups in performed home math activities, but small differences existed in parents’ math-related expectations and attitudes. We found no evidence for associations between children’s home math environment and their numerical and patterning skills. Neither gender nor SES moderated the home math environment – mathematical skills associations.

### Children’s Home Math Environment: Activities, Expectations and Attitudes

The current study revealed substantial variability in children’s home math environment. In line with previous studies (LeFevre et al., 2010; Susperreguy et al., 2020b), parents’ math-related expectations correlated with the frequency of math-related activities such that parents with higher expectations performed more math-related activities at home, and the evidence for this association was decisive. We also observed, similar to Blevins-Knabe et al. (2000), Missall et al. (2015), and Susperreguy et al. (2020b), an association between parents’ attitudes toward mathematics and the math-related activities they performed at home. This association was significant but small (see also Susperreguy et al., 2020a) and the Bayesian analyses indicated that there was neither evidence in favor nor against such an association. The same pattern of findings was observed for the association between parents’ attitudes toward mathematics and their math-related expectations for their children.

### Variability in Children’s Home Math Environment: Effects of Gender and SES

We did not find any evidence for gender differences in children’s home math environment, which is different from what has been observed in Blevins-Knabe and Musun-Miller (1996), Chang et al. (2011), Hart et al. (2016), and del Río et al. (2017). On the other hand, our data are in line with Jordan et al. (2006) and Zippert and Rittle-Johnson (2020), who did not find any gender differences in young children’s home math environment. The Bayesian analyses extend these earlier findings by clearly showing moderate evidence for the null hypothesis of no gender differences. Research of Stoet and Geary (2013) has shown variability in the existence of gender differences in 15-year-olds’ mathematical performances between (mostly high-achieving) countries. Gender differences in mathematics performances, which vary between countries, might be related to differences in young boys’ and girls’ home math environment (see Chang et al., 2011), which may also vary across countries, a possibility that requires further investigation.

Although not the specific focus of the current study, our data further replicate earlier reports of no gender differences in children’s numerical skills at this young age (Kersey et al., 2018; Bakker et al., 2019; Hutchison et al., 2019). The current sample was the same as in Bakker et al. (2019), yet the present study extends their findings by showing that the earlier reported evidence for gender equality in numerical abilities at the age of 4-5 years continues to exist 1 year later at the age of 5-6 years. We further observed a small gender difference favoring girls in patterning ability, although the evidence for this finding was only anecdotal. Similar findings were recently observed by Lüken and Sauzet (2020). These authors suggested that gender differences in children’s early patterning ability might be due to girls engaging more in patterning activities during free play at this age, leading girls to have more experience with these activities. This is a possibility that needs further investigation.

We did not find an effect of children’s SES on the reported math-related activities in their home environment. This is similar to Missall et al. (2015) and Hart et al. (2016). Other researchers have, however, reported positive associations between SES and parent-child math-related activities (Starkey et al., 1999, as cited in Starkey et al., 2004; Jordan et al., 2006; Dearing et al., 2012). One explanation for these conflicting findings might be that such positive associations are only observed for certain types of activities. For example, Susperreguy et al. (2020a) recently found that children’s SES was positively correlated with some math-related activities (i.e., operational activities and shared number-game play) but not with others (i.e., mapping activities). Future studies should investigate differences in the type and frequency of math-related activities that are provided for children stemming from different SES backgrounds.

In line with Susperreguy et al. (2020b), we found that parents from low SES backgrounds had higher math-related expectations for their children than parents from above-average and high SES backgrounds, and that parents from high SES backgrounds had more positive attitudes toward mathematics than parents from above-average or below-average backgrounds. One explanation for the first finding could be that parents of low SES children find it important for their children to master several mathematical skills from an early age onwards, while parents of children with high socioeconomic background believe that these skills will develop automatically in their children when they grow older and, for example, receive more schooling. The more positive attitudes (e.g., I like mathematics) from high SES parents might stem from their own prior experiences with mathematics, as high SES parents may be more likely to have higher mathematical skills, leading to more positive experiences with mathematics. Future research is necessary to provide further insight into these matters, as the present study cannot confirm these hypotheses.

Not unexpectedly, our data revealed strong associations between SES and children’s numerical and patterning skills, with children from higher SES backgrounds showing better performance. This aligns with earlier data on the association between SES and children’s early mathematical competencies (e.g., Jordan and Levine, 2009).

Importantly, it needs to be acknowledged that we operationalized SES only via the educational level of the mother. This was motivated by the fact that parental educational level has been identified as the strongest SES predictor of children’s educational success (Davis-Kean, 2005). The educational level of the mothers in this study was strongly correlated with that of the fathers (*r* = 0.67), a phenomenon known as educational assortative mating (Eika et al., 2019). A *post hoc* analysis of the data moreover indicated that the findings were generally the same when the educational level of the father was used instead of that of the mother.

We would like to emphasize that, despite our deliberate sampling procedure to include diverse SES backgrounds, the number of children in the low SES group was small. Moreover, the educational level of the parents of the children in this group was much more heterogeneous than in the other SES groups: the low SES group comprised parents with widely varying profiles, ranging from no education to lower secondary education degree. We also speculate that the parents who consented to participate in this study, but who did not complete the home math questionnaire (26/379 or 7%), came from lower SES backgrounds, given that children of parents who did return the questionnaire showed significantly better numerical [*t*(378) = −3.42, *p* = 0.001] and patterning abilities [*t*(26.73) = −3.21, *p* = 0.003] (see also Sirin, 2005). This possibility is, however, hard to verify, given that for these parents no information about their educational level was available. This should be kept in mind when considering the generalizability of our findings.

### Associations Between Children’s Home Math Environment and Their Mathematical Skills

We observed little to no associations between children’s home math environment and their numerical and patterning skills. The Bayesian analyses even indicated that there was moderate to strong evidence for the null hypothesis of no association for most of the home math components. These observations align with earlier studies focusing on children’s early mathematical skills (e.g., Blevins-Knabe et al., 2000; Missall et al., 2015; Zippert and Rittle-Johnson, 2020) and children’s spontaneous number focusing tendencies (e.g., Batchelor, 2014; Rathé et al., 2020). They, however, contrast with findings from other studies (e.g., LeFevre et al., 2009; Anders et al., 2012; Dearing et al., 2012; Kleemans et al., 2012; Niklas and Schneider, 2014; Hart et al., 2016; Mutaf-Yildiz et al., 2018a; Susperreguy et al., 2020a) in the same age group that used similar questionnaires to assess the home math environment.

The absence of an association between children’s home math environment and their mathematical ability may be due to the presence of moderating variables, such as gender or SES. We tested this by examining the moderating impact of the latter two variables. These analyses revealed that neither gender nor SES moderated the association between children’s home math environment and their mathematical skills. This lack of moderating effects of gender and SES on the relationship between the home environment and children’s academic achievement aligns with what has been observed in older primary school children (Ciping et al., 2015).

How might the absence of an association between children’s home math environment and their mathematical skills in our study be explained? One explanation relates to the characteristics of the preschool learning environment of the children in the current study. This is a factor that has been relatively ignored in research on the home math environment, despite the evidence showing that the quality of children’s preschool is associated with their early mathematical development (e.g., Anders et al., 2012). Unfortunately, it was not possible to measure and analyze the quality of the preschools in our study, which should be considered as an important limitation. However, we contend that the general context in which the data were collected might explain our results. More specifically, the present study was executed in Flanders (Belgium) were nearly all children (98%) of a particular birth cohort participate in a high-quality preschool system, which is fully subsidized by the government and where parents can freely choose the preschool of their choice (Bos et al., 2016). Preschool education is delivered by trained preschool teachers, who aim for specific learning goals with their children before they transition to the first grade of primary school in the year they turn 6 years. An important part of these learning objectives is focused on children’s mathematical development (e.g., goals related to counting, number, measurement, and patterning). As a result, the current data were collected in a relatively homogeneous preschool education context, where nearly all children are introduced to various mathematical activities from an early age, and several of these activities were included in the administered home math questionnaire. The systematic attention to mathematical activities in this rather homogeneous and open preschool context with a high participation rate might have an impact on the home math environment parents provide. This context might weaken the association between children’s home environment and their early mathematical skills. Such an explanation remains, however, speculative in the absence of cross-cultural studies or studies that are able to compare different preschool systems. Future studies are therefore needed to compare the association between children’s home math environment and their mathematical skills in countries that vary widely in their preschool system and participation rate. Against the background of the current data, we predict that the association between children’s home math environment and their mathematical skills will be weaker in countries with open preschool education systems and high participation rates. In any case, it is critical for future studies on children’s home math environment to also provide explicit information on the preschool system in which children are enrolled. This undoubtedly impacts the home math environment, and consequently its association with children’s mathematical skills, and might explain differences between different studies and countries. Taking into account Vygotsky’s social development theory (1978), and in line with the assumption of the potential impact of children’s preschool on children’s development and outcomes (e.g., Sammons et al., 2008; Anders et al., 2012), it might be possible that young children’s teachers are (the most) important knowledgeable others when it comes to the development of early mathematical skills. This would suggest that it might be critical to aim for high participation rates in preschool from an early age onwards as well as to invest in high-quality preschool systems for all children. It seems therefore important to further investigate the role of children’s preschool and its quality in the development of early mathematical skills, as the present study cannot confirm this hypothesis.

A second reason for the absence of an association between children’s home math environment and their mathematical skills might lie in the use of questionnaire data to investigate children’s home environment. Our measure had good reliability and was highly similar to questionnaires that have been used in previous research that observed associations between children’s home math environment and their mathematical skills (e.g., LeFevre et al., 2009; Kleemans et al., 2012). There are nevertheless several issues that challenge the validity of such questionnaire data. One problem is that parents have to retrospectively report on their activities and that they might not always precisely remember the frequency of these activities. Second, their responses might also be biased due to personal beliefs about their parental role or their intentions, such as spending more time with the child (Green et al., 2007). Third, Likert scales are not always unequivocal to interpret for respondents, and can therefore lead to different interpretations of the provided answer options (Schwarz and Oyserman, 2001). A fourth issue is that the activities that are being questioned are all considered on the same scale of frequency, whereas, in fact, they might vary considerably in their duration and therefore result in different relationships with children’s mathematical skills. For example, playing math-related games or reading picture books are activities that are much more time-consuming than counting the apples during cooking, and are therefore not fully comparable. This requires a fine-grained psychometric item analysis of the different items that are used in home math questionnaires, to determine a score that optimally reflects such differences in the duration of activities (see Purpura et al., 2019). A final, yet related, problem is that information obtained through questionnaires might not capture information about the quality of parent–child interactions. For example, this quality might be determined by the mathematical language parents use when they engage in math-related activities (e.g., Gunderson and Levine, 2011; Ramani et al., 2015; Susperreguy and Davis-Kean, 2016; Elliott et al., 2017). Moreover, according to Vygotsky (1978) it is important to stimulate children’s development by providing activities and practicing skills that are between the child’s own achievement level (problems that can be solved independently) and the level of potential development (problems that can be solved under guidance or in collaboration with more knowledgeable others). Additional support might be necessary for parents to provide high-quality home math activities that are appropriate and meaningful for their children, and that are, as claimed by Vygotsky, within their children’s zone of proximal development (1978). The quality of home math activities is, however, hard to investigate through questionnaires and requires the use of observational methods, which was, unfortunately, not possible in the present study. The association between such observational measures of parent–child math-related interactions and home math questionnaires has been investigated, yet this research revealed that observational data and questionnaire data were not related to each other (Missall et al., 2017; Mutaf-Yildiz et al., 2018b). Observational data on parent–child math-related interactions – when collected in an ecologically valid environment – might provide a more accurate measure of the quality of math-related parent–child activities and interactions. Therefore, these data may provide more fine-grained information on how the home environment impacts on children’s mathematical ability, as compared to research done exclusively by collecting home environment data through questionnaires. From an educational point of view, such research might also suggest which types of interactions are more effective for parents in stimulating their child’s mathematical development. For example, Walsh et al. (2016) showed that asking specific types of questions (i.e., high demanding questions) during shared storybook reading led to improvements in children’s vocabulary, whereas others (i.e., interrupting questions) did not. These findings might also be applicable to the enhancement of children’s mathematical development and this should be investigated in future studies.

## Conclusion

The present study revealed that variability in the parent–child home math activities was not dependent on children’s gender nor SES. Variability in parental math-related expectations and attitudes, however, was related to children’s SES. We found no evidence for associations between 5- to 6-year-old children’s home math environment and their early numerical and patterning skills, and children’s gender or SES did not moderate these relationships. We contend that the educational context in which our investigation was executed might explain these findings. We therefore suggest that future studies should investigate the effects of different educational contexts between as well as within countries to verify the extent to which such differences explain the discrepant findings in the current literature on the relationship between children’s home math environment and their early mathematical skills.

## Data Availability Statement

The raw data supporting the conclusions of this article will be made available by the authors, without undue reservation.

## Ethics Statement

The studies involving human participants were reviewed and approved by the Social and Societal Ethics Committee (SMEC) of KU Leuven. Written informed consent to participate in this study was provided by the participants’ legal guardian/next of kin.

## Author Contributions

LDK: conceptualization, methodology, formal analysis, writing - original draft, and project administration. MB: conceptualization, methodology, formal analysis, and writing - original draft. SR and NW: methodology and writing – review and editing. JT: conceptualization, methodology, writing – review and editing, and project administration. LV: conceptualization, methodology, writing – review and editing, and funding acquisition. BDS: conceptualization, methodology, formal analysis, writing – original draft, and funding acquisition. All authors contributed to the article and aoved the submitted version.

## Conflict of Interest

The authors declare that the research was conducted in the absence of any commercial or financial relationships that could be construed as a potential conflict of interest.

## Footnotes

1 https://ppw.kuleuven.be/o_en_o/CIPenT/wis-co-start

## References

[B1] Agentschap voor Hoger Onderwijs, Volwassenenonderwijs, Kwalificaties en Studietoelagen (n.d.). *Basisonderwijs.* Available online at: *https://onderwijsdoelen.be/resultaten?intro=basisonde-rwijs*&*filters=onderwijsniveau*%*255B0%255D%255Bid%255D%3Df7dcdedc9e-9c97a653c7dba05896ef57a333480b%26onderwijsniveau%255B0%255D%255B-titel%255D%3DBasisonderwijs%26onderwijsniveau%255B0%255D%255Bwa-arde%255D%3DBasisonderwijs%26bo_onderwijs_subniveau%255B0%255D-%255Bid%255D%3D34a77761b6bb8b9e691aec7c5ff36410d430cc6e%26b-o_onderwijs_subniveau%255B0%255D%255Btitel%255D%3DBasisonderwij-s%2520%253E%2520Kleuteronderwijs%26bo_onderwijs_subniveau%255B0-%255D%255Bwaarde%255D%3DKleuteronderwijs* (accessed on 14 January 2020).

[B2] Anders Y. Rossbach H.-G. Weinert S. Ebert S. Kuger S. Lehrl S. (2012). Home and preschool learning environments and their relations to the development of early numeracy skills. *Early Child. Res. Q.* 27 231–244. 10.1016/j.ecresq.2011.08.003

[B3] Andraszewicz S. Scheibehenne B. Rieskamp J. Grasman R. Verhagen J. Wagenmakers E.-J. (2015). An introduction to Bayesian hypothesis testing for management research. *J. Manag.* 41 521–543. 10.1177/0149206314560412

[B4] Bakker M. Torbeyns J. Wijns N. Verschaffel L. De Smedt B. (2019). Gender equality in 4- to 5-year-old preschoolers’ early numerical competencies. *Dev. Sci.* 22:e12718. 10.1111/desc.12718 30175533

[B5] Batchelor S. (2014). *Dispositional Factors Affecting Children’s Early Numerical Development.* PhD dissertation. Loughborough: Loughborough University.

[B6] Blevins-Knabe B. (2016). “Early mathematical development: how the home environment matters,” in *Early Childhood Mathematics Skill Development in the Home Environment*, eds Blevins-Knabe B. Berghout Austin A. (Cham: Springer International Publishing), 7–28. 10.1007/978-3-319-43974-7_2

[B7] Blevins-Knabe B. Berghout Austin A. (eds) (2016). *Early Childhood Mathematics Skill Development in the Home Environment.* Cham: Springer International Publishing.

[B8] Blevins-Knabe B. Berghout Austin A. Musun L. Eddy A. Jones R. M. (2000). Family home care providers’ and parents’ beliefs and practices concerning mathematics with young children. *Early Child. Dev. Care* 165 41–58. 10.1080/0300443001650104

[B9] Blevins-Knabe B. Musun-Miller L. (1996). Number use at home by children and their parents and its relationship to early mathematical performance. *Early Dev. Parent.* 5 35–45. 10.1002/(sici)1099-0917(199603)5:1<35::aid-edp113<3.0.co;2-0

[B10] Bos J. M. Phillips-Fain G. Rein E. Weinberg E. Chavez S. (2016). *Connecting all Children to High-Quality Early Care and Education: Promising Strategies From the International Community.* Washington, DC: American Institutes of Research.

[B11] Calvo A. Bialystok E. (2014). Independent effects of bilingualism and socioeconomic status on language ability and executive functioning. *Cognition* 130 278–288. 10.1016/j.cognition.2013.11.015 24374020 PMC3921957

[B12] Chang A. Sandhofer C. M. Brown C. S. (2011). Gender biases in early number exposure to preschool-aged children. *J. Lang. Soc. Psychol.* 30 440–450. 10.1177/0261927X11416207

[B13] Ciping D. Silinskas G. Wei W. Georgiou G. K. (2015). Cross-lagged relationships between home learning environment and academic achievement in Chinese. *Early Child. Res. Q.* 33 12–20. 10.1016/j.ecresq.2015.05.001

[B14] Clements D. H. Sarama J. (2014). “Other content domains,” in *Learning and Teaching Early Math: The Learning Trajectories Approach*, eds Clements D. H. Sarama J. (New York, NY: Routledge), 214–229.

[B15] Davis-Kean P. E. (2005). The influence of parent education and family income on child achievement: the indirect role of parental expectations and the home environment. *J. Fam. Psychol.* 19 294–304. 10.1037/0893-3200.19.2.294 15982107

[B16] Dearing E. Casey B. M. Ganley C. M. Tillinger M. Laski E. Montecillo C. (2012). Young girls’ arithmetic and spatial skills: the distal and proximal roles of family socioeconomics and home learning experiences. *Early Child. Res. Q.* 27 458–470. 10.1016/j.ecresq.2012.01.002

[B17] del Río M. F. Susperreguy M. I. Strasser K. Salinas V. (2017). Distinct influences of mothers and fathers on kindergartners’ numeracy performance: the role of math anxiety, home numeracy practices, and numeracy expectations. *Early Educ. Dev.* 28 939–955. 10.1080/10409289.2017.1331662

[B18] Duncan G. J. Dowsett C. J. Claessens A. Magnuson K. Huston A. C. Klebanov P. (2007). School readiness and later achievement. *Dev. Psychol.* 43 1428–1446. 10.1037/0012-1649.43.6.1428 18020822

[B19] Eccles J. S. (1993). “School and family effects on the ontogeny of children’s interests, self-perceptions, and activity choices,” in *Nebraska Symposium on Motivation, 1992: Developmental Perspectives on Motivation*, ed. Jacobs J. E. (Lincoln, NE: University of Nebraska Press), 145–208. 1340520

[B20] Economist Intelligence Unit (2012). *Starting Well: Benchmarking Early Education Across the World.* London: The Economist Intelligence Unit.

[B21] Eika L. Mogstad M. Zafar B. (2019). Educational assortative mating and household income inequality. *J. Polit. Econ.* 127 2795–2835. 10.1086/702018

[B22] Elliott L. Braham E. J. Libertus M. E. (2017). Understanding sources of individual variability in parents’ number talk with young children. *J. Exp. Child Psychol.* 159 1–15. 10.1016/j.jecp.2017.01.011 28266331

[B23] Green C. L. Walker J. M. T. Hoover-Dempsey K. V. Sandler H. M. (2007). Parents’ motivations for involvement in children’s education: an empirical test of a theoretical model of parental involvement. *J. Educ. Psychol.* 99 532–544. 10.1037/0022-0663.99.3.532

[B24] Gunderson E. A. Levine S. C. (2011). Some types of parent number talk count more than others: relations between parents’ input and children’s cardinal-number knowledge. *Dev. Sci.* 14 1021–1032. 10.1111/j.1467-7687.2011.01050.x 21884318 PMC3177161

[B25] Hart S. A. Ganley C. M. Purpura D. J. (2016). Understanding the home math environment and its role in predicting parent report of children’s math skills. *PLoS One* 11:e0168227. 10.1371/journal.pone.0168227 28005925 PMC5179117

[B26] Hayes A. F. (2017). *Introduction to Mediation, Moderation, and Conditional Process Analysis: A Regression-Based Approach*, 2nd Edn New York, NY: The Guilford Press.

[B27] Hayes A. F. (2020). *PROCESS Macro (Version 3.5). Computer software.*

[B28] Huntsinger C. S. Jose P. E. Larson S. L. Balsink Krieg D. Shaligram C. (2000). Mathematics, vocabulary, and reading development in Chinese American and European American children over the primary school years. *J. Educ. Psychol.* 92 745–760. 10.1037//0022-0663.92.4.745

[B29] Hutchison J. E. Lyons I. M. Ansari D. (2019). More similar than different: gender differences in children’s basic numerical skills are the exception not the rule. *Child Dev.* 90 e66–e79. 10.1111/cdev.13044 29484644

[B30] Hyde J. S. Fennema E. Lamon S. J. (1990). Gender differences in mathematics performance: a meta-analysis. *Psychol. Bull.* 107 139–155. 10.1037//0033-2909.107.2.139 2138794

[B31] IBM Corp. (2017). *SPSS statistics (Version 25). Computer software.* Armonk, NY: IBM Corp.

[B32] IBM Corp. (2019). *SPSS statistics (Version 26). Computer software.* Armonk, NY: IBM Corp.

[B33] JASP Team (2019). *JASP (Version 0.11*.1). Computer software.

[B34] Jordan N. C. Kaplan D. Nabors Oláh L. Locuniak M. N. (2006). Number sense growth in kindergarten: a longitudinal investigation of children at risk for mathematics difficulties. *Child Dev.* 77 153–175. 10.1111/j.1467-8624.2006.00862.x 16460531

[B35] Jordan N. C. Levine S. C. (2009). Socioeconomic variation, number competence, and mathematics learning difficulties in young children. *Dev. Disabil. Res. Rev.* 15 60–68. 10.1002/ddrr.46 19213011

[B36] Kersey A. J. Braham E. J. Csumitta K. D. Libertus M. E. Cantlon J. F. (2018). No intrinsic gender differences in children’s earliest numerical abilities. *NPJ Sci. Learn.* 3 1–10. 10.1038/s41539-018-0028-7 30631473 PMC6220191

[B37] Kleemans T. Peeters M. Segers E. Verhoeven L. (2012). Child and home predictors of early numeracy skills in kindergarten. *Early Child. Res. Q.* 27 471–477. 10.1016/j.ecresq.2011.12.004

[B38] Klein A. Starkey P. (2004). “Fostering preschool children’s mathematical knowledge: findings from the Berkeley math readiness project,” in *Engaging Young Children in Mathematics: Standards for Early Childhood Mathematics in Education*, eds Clements D. H. Sarama J. DiBiase A.-M. (Mahwah, NJ: Lawrence Erlbaum Associates), 343–360.

[B39] LeFevre J.-A. Polyzoi E. Skwarchuk S.-L. Fast L. Sowinski C. (2010). Do home numeracy and literacy practices of Greek and Canadian parents predict the numeracy skills of kindergarten children? *Int. J. Early Years Educ.* 18 55–70. 10.1080/09669761003693926

[B40] LeFevre J.-A. Skwarchuk S.-L. Smith-Chant B. L. Fast L. Kamawar D. Bisanz J. (2009). Home numeracy experiences and children’s math performance in the early school years. *Can. J. Behav. Sci.* 41 55–66. 10.1037/a0014532

[B41] Lindberg S. M. Hyde J. S. Petersen J. L. Linn M. C. (2010). New trends in gender and mathematics performance: a meta-analysis. *Psychol. Bull.* 136 1123–1135. 10.1037/a0021276 21038941 PMC3057475

[B42] Lüken M. M. Sauzet O. (2020). Patterning strategies in early childhood: a mixed methods study examining 3- to 5-year-old children’s patterning competencies. *Math. Think. Learn.* 10.1080/10986065.2020.1719452 [Epub ahead of print].

[B43] Marks G. N. McMillan J. Jones F. L. Ainley J. (2000). *The Measurement of Socioeconomic Status for the Reporting of Nationally Comparable Outcomes of Schooling.* Camberwell, VI: Australian Council for Educational Research.

[B44] Missall K. Hojnoski R. L. Caskie G. I. L. Repasky P. (2015). Home numeracy environments of preschoolers: examining relations among mathematical activities, parent mathematical beliefs, and early mathematical skills. *Early Educ. Dev.* 26 356–376. 10.1080/10409289.2015.968243

[B45] Missall K. N. Hojnoski R. L. Moreano G. (2017). Parent–child mathematical interactions: examining self-report and direct observation. *Early Child Dev. Care* 187 1896–1908. 10.1080/03004430.2016.1193731

[B46] Mutaf-Yildiz B. Sasanguie D. De Smedt B. Reynvoet B. (2018a). Frequency of home numeracy activities is differentially related to basic number processing and calculation skills in kindergartners. *Front. Psychol.* 9:340. 10.3389/fpsyg.2018.00340 29623055 PMC5874519

[B47] Mutaf-Yildiz B. Sasanguie D. De Smedt B. Reynvoet B. (2018b). Investigating the relationship between two home numeracy measures: a questionnaire and observations during lego building and book reading. *Br. J. Dev. Psychol.* 36 354–370. 10.1111/bjdp.12235 29393519

[B48] Napoli A. R. Purpura D. J. (2018). The home literacy and numeracy environment in preschool: cross-domain relations of parent–child practices and child outcomes. *J. Exp. Child Psychol.* 166 581–603. 10.1016/j.jecp.2017.10.002 29102840

[B49] Niklas F. Schneider W. (2014). Casting the die before the die is cast: the importance of the home numeracy environment for preschool children. *Eur. J. Psychol. Educ.* 29 327–345. 10.1007/s10212-013-0201-6

[B50] Purpura D. J. Borriello G. Schmitt S. A. (2019). “Item-level variability in the assessment of the home numeracy environment: a graded response model analysis,” in *Proceedings of the Symposium Conducted at the Annual Meeting of the Mathematical Learning and Cognition Society on V. Simms (chair) A Tricky Mathematical Problem: Developing Rigorous and Valid Measurements of the Preschool Home Numeracy Environment*, Ottawa, ON.

[B51] Purpura D. J. Lonigan C. J. (2013). Informal numeracy skills: the structure and relations among numbering, relations, and arithmetic operations in preschool. *Am. Educ. Res. J.* 50 178–209. 10.3102/0002831212465332

[B52] Ramani G. B. Rowe M. L. Eason S. H. Leech K. A. (2015). Math talk during informal learning activities in head start families. *Cogn. Dev.* 35 15–33. 10.1016/j.cogdev.2014.11.002

[B53] Rathé S. Torbeyns J. De Smedt B. Verschaffel L. (2020). Are children’s spontaneous number focusing tendencies related to their home numeracy environment? *ZDM* 52 729–742. 10.1007/s11858-020-01127-z

[B54] Rittle-Johnson B. Fyfe E. R. Loehr A. M. Miller M. R. (2015). Beyond numeracy in preschool: adding patterns to the equation. *Early Child. Res. Q.* 31 101–112. 10.1016/j.ecresq.2015.01.005

[B55] Sammons P. Anders Y. Sylva K. Melhuish E. Siraj-Blatchford I. Taggart B. (2008). Children’s cognitive attainment and progress in English primary schools during key stage 2: investigating the potential continuing influences of pre-school education. *ZfE* 10 179–198. 10.1007/978-3-531-91452-7

[B56] Schwarz N. Oyserman D. (2001). Asking questions about behavior: cognition, communication, and questionnaire construction. *AJE* 22 127–160. 10.1016/S1098-2140(01)00133-3

[B57] Segers E. Kleemans T. Verhoeven L. (2015). Role of parent literacy and numeracy expectations and activities in predicting early numeracy skills. *Math. Think. Learn.* 17 219–236. 10.1080/10986065.2015.1016819

[B58] Sirin S. R. (2005). Socioeconomic status and academic achievement: a meta-analytic review of research. *Rev. Educ. Res.* 75 417–453. 10.3102/00346543075003417

[B59] Skwarchuk S.-L. (2009). How do parents support preschoolers’ numeracy learning experiences at home? *Early Child Educ. J.* 37 189–197. 10.1007/s10643-009-0340-1

[B60] Skwarchuk S.-L. Sowinski C. LeFevre J.-A. (2014). Formal and informal home learning activities in relation to children’s early numeracy and literacy skills: the development of a home numeracy model. *J. Exp. Child Psychol.* 121 63–84. 10.1016/j.jecp.2013.11.006 24462995

[B61] Starkey P. Klein A. Wakeley A. (2004). Enhancing young children’s mathematical knowledge through a pre-kindergarten mathematics intervention. *Early Child. Res. Q.* 19 99–120. 10.1016/j.ecresq.2004.01.002

[B62] Stoet G. Geary D. C. (2013). Sex differences in mathematics and reading achievement are inversely related: within- and across-nation assessment of 10 Years of PISA data. *PLoS One* 8:e57988. 10.1371/journal.pone.0057988 23516422 PMC3596327

[B63] Susperreguy M. I. Davis-Kean P. E. (2016). Maternal math talk in the home and math skills in preschool children. *Early Educ. Dev.* 27 841–857. 10.1080/10409289.2016.1148480

[B64] Susperreguy M. I. Di Lonardo, Burr S. Xu C. Douglas H. LeFevre J.-A. (2020a). Children’s home numeracy environment predicts growth of their early mathematical skills in kindergarten. *Child Dev.* 91 1663–1680. 10.1111/cdev.13353 31960956

[B65] Susperreguy M. I. Douglas H. Xu C. Molina-Rojas N. LeFevre J.-A. (2020b). Expanding the home numeracy model to Chilean children: relations among parental expectations, attitudes, activities, and children’s mathematical outcomes. *Early Child. Res. Q.* 50 16–28. 10.1016/j.ecresq.2018.06.010

[B66] UCLA Statistical Consulting Group (2020). *Coding Systems for Categorical Variables in Regression Analysis.* Available online at: *https://stats.idre.ucla.edu/spss/faq/coding-systems-for-categorical-variables-in-regression-analysis-*2/ (accessed on September 15, 2020).

[B67] Vygotsky L. S. (1978). *Mind in Society: The Development of Higher Psychological Processes.* Cambridge: Harvard University Press.

[B68] Walsh B. A. Sanchez C. Burnham M. M. (2016). Shared storybook reading in head start: impact of questioning styles on the vocabulary of Hispanic dual language learners. *Early Child. Educ. J.* 44 263–273. 10.1007/s10643-015-0708-3

[B69] Wijns N. De Smedt B. Verschaffel L. Torbeyns J. (2019a). Are preschoolers who spontaneously create patterns better in mathematics? *Br. J. Educ. Psychol.* 90 753–769. 10.1111/bjep.12329 31814113

[B70] Wijns N. Torbeyns J. Bakker M. De Smedt B. Verschaffel L. (2019b). Four-year olds’ understanding of repeating and growing patterns and its association with early numerical ability. *Early Child. Res. Q.* 49 152–163. 10.1016/j.ecresq.2019.06.004

[B71] Zippert E. L. Rittle-Johnson B. (2020). The home math environment: more than numeracy. *Early Child. Res. Q.* 50 4–15. 10.1016/j.ecresq.2018.07.009

