# Supplementary material for: No Association Between the Home Math Environment and Numerical and Patterning Skills in a Large and Diverse Sample of 5- to 6-year-olds
Source: Front Psychol. 2020 Dec 10;11:547626. doi: 10.3389/fpsyg.2020.547626 (PMC7758193; doi:10.3389/fpsyg.2020.547626)
Supplement: Supplementary file 1 [file Table_1.pdf]

Appendix A

Home math environment questionnaire

Home math activities

How often do you engage in the following activities together with your child? *(Cross 1 box that correctly indicates your answer)*

	Never	Less than once a week	Once a week	More than once a week	Every day
Playing games that require counting or elementary computations (e.g., Snakes and ladders; Jungle game)	☐	☐	☐	☐	☐
Reading picture books that include numbers, counting or elementary computations (e.g., Count with Maisy)	☐	☐	☐	☐	☐
Singing counting rhymes or counting songs (e.g., 5 little monkeys)	☐	☐	☐	☐	☐
Attending to written numerals during daily activities (e.g., cooking)	☐	☐	☐	☐	☐
Counting or elementary computations during daily activities (e.g., counting the number of apples during cooking)	☐	☐	☐	☐	☐
Creating patterns with concrete materials (e.g., creating a necklace with alternating red and blue beads)	☐	☐	☐	☐	☐
Playing games that include patterns (e.g., Team umizoomi)	☐	☐	☐	☐	☐

Parental expectations

How important is it for you that your child masters the following competencies at the start of first grade? *(Cross 1 box that correctly indicates your answer)*

	Not at all important	Not important	Important	Very important
Reciting the number sequence up to 10 (e.g., 1, 2, 3, 4, …)	☐	☐	☐	☐
Reciting the number sequence over 10 (e.g., 10, 11, 12, 13, …)	☐	☐	☐	☐
Counting up to 10 objects (e.g., counting 3 candies)	☐	☐	☐	☐
Counting more than 10 objects (e.g., counting 12 cubes)	☐	☐	☐	☐
Reading written number symbols (e.g., 3)	☐	☐	☐	☐
Writing number symbols (e.g., 2)	☐	☐	☐	☐
Solving sums up to 5 (e.g., 2 + 2)	☐	☐	☐	☐
Solving sums up to 10 (e.g., 5 + 4)	☐	☐	☐	☐
Extending a pattern (e.g., red blue red blue)	☐	☐	☐	☐
Naming or describing a pattern	☐	☐	☐	☐

Parental attitudes

What is your attitude toward mathematics? *(Cross 1 box that correctly indicates your answer)*

	Completely disagree	Disagree	Neutral	Agree	Completely agree
I like mathematics	☐	☐	☐	☐	☐
Mathematics is important	☐	☐	☐	☐	☐
I am competent in mathematics	☐	☐	☐	☐	☐
