# Supplementary material for: No Association Between the Home Math Environment and Numerical and Patterning Skills in a Large and Diverse Sample of 5- to 6-year-olds
Source: Front Psychol. 2020 Dec 10;11:547626. doi: 10.3389/fpsyg.2020.547626 (PMC7758193; doi:10.3389/fpsyg.2020.547626)
Supplement: Supplementary file 2 [file Table_2.pdf]

Appendix B

Descriptive statistics of the home math environment questionnaire

Scale and items	*n*	*M*	*Min*	*Max*	*SD*
Home math environment – activities[a]					
Playing games that require counting or elementary computations (e.g., Snakes and ladders; Jungle game)	348	1.93	0.00	4.00	0.95
Reading picture books that include numbers, counting or elementary computations (e.g., Count with Maisy)	349	1.84	0.00	4.00	1.10
Singing counting rhymes or counting songs (e.g., 5 little monkeys)	351	1.65	0.00	4.00	1.07
Attending to written numerals during daily activities (e.g., cooking)	349	2.13	0.00	4.00	1.13
Counting or elementary calculations during daily activities (e.g., counting the number of apples during cooking)	349	2.57	0.00	4.00	1.00
Creating patterns with concrete materials (e.g., creating a necklace with alternating red and blue beads)	347	1.17	0.00	4.00	0.97
Playing games that include patterns (e.g., Team umizoomi)	346	1.36	0.00	4.00	1.05
Home math environment – expectations[b]					
Reciting the number sequence up to 10 (e.g., 1, 2, 3, 4, …)	344	2.68	1.00	3.00	0.53
Reciting the number sequence over 10 (e.g., 10, 11, 12, 13, …)	345	2.19	0.00	3.00	0.74
Counting up to 10 objects (e.g., counting 3 candies)	345	2.57	1.00	3.00	0.57

Counting more than 10 objects (e.g., counting 12 cubes)	346	2.12	0.00	3.00	0.76
Reading written number symbols (e.g., 3)	345	2.11	0.00	3.00	0.78
Writing number symbols (e.g., 2)	347	1.67	0.00	3.00	0.85
Solving sums up to 5 (e.g., 2 + 2)	342	1.52	0.00	3.00	0.92
Solving sums up to 10 (e.g., 5 + 4)	343	1.23	0.00	3.00	0.90
Extending a pattern (e.g., red blue red blue	345	2.25	0.00	3.00	0.62
Naming or describing a pattern	342	1.94	0.00	3.00	0.78
Home math environment – attitudes [c]					
I like mathematics	347	2.71	0.00	4.00	1.08
Mathematics is important	346	3.47	0.00	4.00	0.67
I am competent in mathematics	346	2.58	0.00	4.00	1.02

[a] Rated on a 0-4 rating scale, with 0 = never, 4 = every day; [b] Rated on a 0-3 rating scale, with 0 = not at all important, 3 = very important; [c] Rated on a 0-4 rating scale, with 0 = completely disagree, 4 = completely agree.
