# Supplementary material for: No Association Between the Home Math Environment and Numerical and Patterning Skills in a Large and Diverse Sample of 5- to 6-year-olds
Source: Front Psychol. 2020 Dec 10;11:547626. doi: 10.3389/fpsyg.2020.547626 (PMC7758193; doi:10.3389/fpsyg.2020.547626)
Supplement: Supplementary file 3 [file Table_3.pdf]

Appendix C

Table C1

Moderation analyses of the association between activities, children's gender and their numerical skills (F(3, 347) = 0.85, p = .470, R^2 = .01)

	B	*SE*	*t*	*p*
Activities	0.04	0.07	0.57	.569
Gender	-0.10	0.07	-1.44	.150
Activities*Gender	-0.06	0.11	-0.61	.545

Table C2

Moderation analyses of the association between expectations, children's gender and their numerical skills (F(3, 343) = 1.95, p = .121, R^2 = .02)

	B	*SE*	*t*	*p*
Expectations	0.17	0.09	1.78	.076
Gender	-0.10	0.07	-1.38	.168
Expectations*Gender	-0.25	0.14	-1.84	.067

Table C3

Moderation analyses of the association between attitudes, children's gender and their numerical skills (F(3, 344) = 3.03, p = .029, R^2 = .03)

	B	*SE*	*t*	*p*
Attitudes	0.12	0.07	1.72	.086
Gender	-0.12	0.07	-1.59	.113
Attitudes *Gender	0.00	0.09	-0.01	.990

Table C4

Moderation analyses of the association between activities, SES and children's numerical skills

($F(7, 338) = 3.94$, $p < .001$, $R^2 = .08$)

	B	*SE*	*t*	*p*
Activities	0.02	0.05	0.34	.738
SES-1 (1 vs. 2, 3, 4)	0.31	0.11	2.70	.007
SES-2 (2 vs. 3, 4)	0.33	0.08	3.96	<.001
SES-3 (3 vs. 4)	0.11	0.09	1.18	.240
Activities*SES-1 (1 vs. 2, 3, 4)	0.11	0.13	0.85	.398
Activities*SES-2 (2 vs. 3, 4)	-0.09	0.12	-0.75	.454
Activities*SES-3 (3 vs. 4)	-0.05	0.14	-0.33	.744

Note. Analyses were performed using Helmert coding (1 = low SES; 2 = below-average SES; 3 = above-average SES; 4 = high SES).

Table C5

Moderation analyses of the association between expectations, SES and children's numerical skills ($F(7, 334) = 4.70$, $p < .001$, $R^2 = .09$)

	B	*SE*	*t*	*p*
Expectations	0.12	0.08	1.61	.11
SES-1 (1 vs. 2, 3, 4)	0.34	0.12	2.82	.01
SES-2 (2 vs. 3, 4)	-0.28	0.17	-1.67	.10
SES-3 (3 vs. 4)	0.10	0.18	0.58	.56
Expectations*SES-1 (1 vs. 2, 3, 4)	0.03	0.22	0.12	.90
Expectations*SES-2 (2 vs. 3, 4)	-0.28	0.17	-1.67	.10
Expectations*SES-3 (3 vs. 4)	0.10	0.18	0.58	.56

Note. Analyses were performed using Helmert coding (1 = low SES; 2 = below-average SES; 3 = above-average SES; 4 = high SES).

Table C6

Moderation analyses of the association between attitudes, SES and children's numerical skills

($F(7, 335) = 5.07$, $p < .001$, $R^2 = .10$)

	B	*SE*	*t*	*p*
Attitudes	0.12	0.06	2.17	.031
SES-1 (1 vs. 2, 3, 4)	0.38	0.11	3.34	<.001
SES-2 (2 vs. 3, 4)	0.30	0.08	3.52	<.001
SES-3 (3 vs. 4)	0.10	0.10	1.04	.300
Attitudes*SES-1 (1 vs. 2, 3, 4)	-0.14	0.18	-0.80	.424
Attitudes*SES-2 (2 vs. 3, 4)	-0.07	0.11	-0.63	.527
Attitudes*SES-3 (3 vs. 4)	-0.15	0.12	-1.31	.192

Note. Analyses were performed using Helmert coding (1 = low SES; 2 = below-average SES; 3 = above-average SES; 4 = high SES).

Table C7

Moderation analyses of the association between activities, children's gender and their patterning skills ($F(3, 347) = 2.90$, $p = .035$, $R^2 = .02$)

	B	*SE*	*t*	*p*
Activities	-0.08	0.08	-1.05	.294
Gender	0.19	0.08	2.47	.014
Activities*Gender	-0.01	0.11	-0.05	.963

Table C8

Moderation analyses of the association between expectations, children's gender and their patterning skills ($F(3, 343) = 2.18$, $p = .090$, $R^2 = .02$)

	B	*SE*	*t*	*p*
Expectations	0.01	0.10	0.07	.947
Gender	0.19	0.08	2.35	.020
Expectations*Gender	-0.11	0.15	-0.72	.474

Table C9

Moderation analyses of the association between attitudes, children's gender and their patterning skills ($F(3, 344) = 2.45$, $p = .063$, $R^2 = .02$)

	B	*SE*	*t*	*p*
Attitudes	-0.06	0.08	-0.82	.412
Gender	0.19	0.08	2.36	.019
Attitudes *Gender	0.14	0.10	1.33	.184

Table C10

Moderation analyses of the association between activities, SES and children's patterning skills

($F(7, 338) = 3.80$, $p < .001$, $R^2 = .07$)

	B	*SE*	*t*	*p*
Activities	-0.10	0.06	-1.74	.084
SES-1 (1 vs. 2, 3, 4)	0.31	0.12	2.57	.011
SES-2 (2 vs. 3, 4)	0.31	0.09	3.52	<.001
SES-3 (3 vs. 4)	0.05	0.10	0.53	.600
Activities*SES-1 (1 vs. 2, 3, 4)	0.16	0.15	1.12	.264
Activities*SES-2 (2 vs. 3, 4)	0.19	0.13	1.40	.162
Activities*SES-3 (3 vs. 4)	-0.08	0.15	-0.55	.585

Note. Analyses were performed using Helmert coding (1 = low SES; 2 = below-average SES; 3 = above-average SES; 4 = high SES).

Table C11

Moderation analyses of the association between expectations, SES and children's patterning skills (F(7, 334) = 3.25, p = .002, R^2 = .06)

	B	*SE*	*t*	*p*
Expectations	0.02	0.09	0.25	.805
SES-1 (1 vs. 2, 3, 4)	0.33	0.13	2.43	.016
SES-2 (2 vs. 3, 4)	0.35	0.09	3.76	<.001
SES-3 (3 vs. 4)	0.06	0.10	0.55	.583
Expectations*SES-1 (1 vs. 2, 3, 4)	-0.10	0.24	-0.40	.694
Expectations*SES-2 (2 vs. 3, 4)	-0.01	0.19	-0.06	.955
Expectations*SES-3 (3 vs. 4)	0.04	0.20	0.22	.824

Note. Analyses were performed using Helmert coding (1 = low SES; 2 = below-average SES; 3 = above-average SES; 4 = high SES).

Table C12

Moderation analyses of the association between attitudes, SES and children's patterning skills

(F(7, 335) = 3.51, p = .001, R^2 = .07

	B	*SE*	*t*	*p*
Attitudes	0.00	0.06	0.04	.967
SES-1 (1 vs. 2, 3, 4)	0.33	0.12	2.67	.008
SES-2 (2 vs. 3, 4)	0.36	0.09	3.84	<.001
SES-3 (3 vs. 4)	0.06	0.10	0.54	.591
Attitudes*SES-1 (1 vs. 2, 3, 4)	-0.12	0.20	-0.62	.538
Attitudes*SES-2 (2 vs. 3, 4)	0.13	0.12	1.08	.282
Attitudes*SES-3 (3 vs. 4)	-0.13	0.13	-1.03	.302

Note. Analyses were performed using Helmert coding (1 = low SES; 2 = below-average SES; 3 = above-average SES; 4 = high SES).
